# The Effect of Own Body Concerns on Judgments of Other Women’s Body Size

**DOI:** 10.3389/fpsyg.2022.888904

**Published:** 2022-05-06

**Authors:** Katri K. Cornelissen, Lise Gulli Brokjøb, Jiří Gumančík, Ellis Lowdon, Kristofor McCarty, Kamila R. Irvine, Martin J. Tovée, Piers Louis Cornelissen

**Affiliations:** ^1^Department of Psychology, Northumbria University, Newcastle upon Tyne, United Kingdom; ^2^Department of Psychology, UiT The Arctic University of Norway, Tromsø, Norway; ^3^School of Psychology, College of Social Science, University of Lincoln, Lincolnshire, United Kingdom

**Keywords:** self-estimated body size, body image dissatisfaction, BMI, anorexia nervosa, social comparison, thin ideal

## Abstract

We investigated the relationships between healthy women’s estimates of their own body size, their body dissatisfaction, and how they subjectively judge the transition from normal to overweight in other women’s bodies (the “normal/overweight” boundary). We propose two complementary hypotheses. In the first, participants compare other women to an internalized Western “thin ideal,” whose size reflects the observer’s own body dissatisfaction. As dissatisfaction increases, so the size of their “thin ideal” reduces, predicting an inverse relationship between the “normal/overweight” boundary and participants’ body dissatisfaction. Alternatively, participants judge the size of other women relative to the body size they believe they have. For this implicit or explicit social comparison, the participant selects a “normal/overweight” boundary that minimizes the chance of her making an upward social comparison. So, the “normal/overweight” boundary matches or is larger than her own body size. In an online study of 129 healthy women, we found that both opposing factors explain where women place the “normal/overweight” boundary. Increasing body dissatisfaction leads to slimmer judgments for the position of the “normal/overweight” boundary in the body mass index (BMI) spectrum. Whereas, increasing overestimation by the observer of their own body size shifts the “normal/overweight” boundary toward higher BMIs.

## Introduction

Imagine someone who, in your opinion, had a body mass index (BMI) in the normal range, but who is now putting on weight. Subjectively, at what point would you describe them as having crossed over from normal weight to being overweight? What perceptual and attitudinal factors determine where you place this boundary? In this study, we investigated the relationships between women’s estimates of their own body size, their own body dissatisfaction, and how they subjectively judge the transition from normal to overweight in other women’s bodies (the “normal/overweight” boundary). We propose two hypotheses to describe these inter-relationships, both of which depend on a combination of sociocultural theories for body dissatisfaction together with the observer’s point of view. In the first case, we propose that an observer’s judgment about where to set the boundary is made by comparison to their own internalized representation of Western societies ideal of attractiveness, the so-called “thin ideal” (Thompson and Stice, 2001). This constitutes a comparison between two third parties, one of whom resides in the mind of the observer and the other the stimulus viewed. In the second case, we propose that the observer’s judgment is based on a social comparison between the body size they believe themselves to have and the body of the woman in the stimulus image; that is, a comparison between the self and a third party. The aim of this study, therefore, is to ask whether either hypothesis is supported, but we acknowledge that the evidence may support neither hypothesis or both.

### Hypothesis 1: A Comparison Between Two Third Parties

Sociocultural theories, such as the Tripartite Influence Model (Thompson et al., 1999) and Dual Pathway Model (Stice and Agras, 1998), offer powerful explanations for why women in Western society experience concern about their body image. They propose that variable combinations of pressures exerted by media, family, and peers, lead to women becoming dissatisfied with their own bodies (Powell and Kahn, 1995; Levine and Smolak, 1996; Thompson et al., 1999; Stice, 2001; Stice et al., 2001; Thompson and Stice, 2001; Sypeck et al., 2006). The focal point for these pressures is the concept of a “thin ideal” female, frequently promulgated by Western media. As a result, not only are strong cultural associations forged between thinness, attractiveness, desirability, and social status, but the required levels of thinness are also unachievable for most individuals (Hebl and Heatherton, 1998; Evans, 2003). Empirically, a number of experimental studies have shown that short term exposure to Western idealized images of women both induces and enhances body dissatisfaction (see, e.g., Becker et al., 2002), and this conclusion is supported by meta-analyses (Groesz et al., 2001). In addition, the extent to which women internalize the Western “thin ideal” seems to predict body dissatisfaction (Stice, 1994, 2002; Thompson and Stice, 2001; Stice et al., 2003). Conversely, women who do not follow this path are less likely to develop body dissatisfaction and eating disorders (Furnham and Alibhai, 1983; Pate et al., 1992; Akan and Grilo, 1995).

Critically, a number of authors have used photorealistic 3D avatars or line drawings to show that both women’s ideal body size, as well as the body size they consider to be normal, is inversely related to their own body dissatisfaction (e.g., Williamson et al., 1993; Glauert et al., 2009). Equivalent results have been obtained using Relational Responding Tasks (RRT) to measure implicit beliefs about actual and desired physical appearance (De Houwer et al., 2015; Heider et al., 2018). Therefore, if we assume that women use these internal representations as a yardstick to judge others, there should be a direct relationship between the magnitude of an observer’s body dissatisfaction and the body size they select to represent the “normal/overweight” boundary for the stimulus: as their own body size dissatisfaction increases, so the “normal/overweight” boundary should decrease. We also assume that the size of the “thin ideal” is not directly related to the body size/shape that the observer has (*cf.* Heider et al., 2018). Therefore, the predicted negative relationship between the “normal/overweight” boundary and the observer’s own body dissatisfaction should also be independent of their actual body size/shape.

### Hypothesis 2: A Comparison Between the Self and a Third Party

Mechanistically, the Tripartite Influence Model shows how direct influences from peer, parental, and media factors, together with mediational links *via* internalization of societal appearance standards and appearance comparison processes lead to body dissatisfaction and eating disturbance (Thompson et al., 1999; Shroff and Thompson, 2006). It is the internalization processes incorporating the “thin ideal” which is central to hypothesis 1. The appearance comparison processes give rise to hypothesis 2. Specifically, when asked to set the “normal/overweight” boundary on another woman’s body, the observer could make this judgment in relation to the body size they think they have themselves, and in so doing, would make either an explicit or implicit social comparison (Festinger, 1954; van den Berg et al., 2002). We suggest that any comparison should either be neutral or downward, because she selects a size for the normal/overweight boundary that is the same or larger than herself. The observer is unlikely to select a boundary that is smaller than she believes herself to be, because this would represent an upward social comparison, and has the potential to cause distress. In other words, in this scenario, the “normal/overweight” boundary should either equate to the body size an observer believes she has or be larger than this. It can be likened to a strategy of size selection that nulls out any potential distress caused by social comparison.

Previous studies have suggested that people tend to make social comparisons which result in positive outcome for themselves (i.e., in this case a downward social comparison; Morrison et al., 2004). However, it is possible that an upward social comparison could occur. Some studies have suggested in appearance judgments there may be a tendency for upward comparison (O’Brien et al., 2009; Fitzsimmons-Craft et al., 2012). But the judgment made in this study is specifically body size, and we propose that it is more likely that our participants will be making a neutral, or downward comparison.

This hypothesis raises the question of what determines the body size a woman believes she has. We know from a number of recent studies using CGI (computer-generated imagery) avatars (Cornelissen, et al., 2015, 2016, 2017; Irvine et al., 2018) that this is determined by two statistically independent factors: (a) perceptual contraction bias and (b) psychological concerns about her body shape, weight, eating, tendency toward depression and self-esteem (*cf.* perceptual versus attitudinal body image, Cash and Deagle, 1997). Contraction bias arises when one uses a standard reference or template for a particular kind of object against which to estimate the size of other examples of that object (Poulton, 1989). The estimate is most accurate when judging the size of an object of a similar size to the reference but becomes increasingly inaccurate as the magnitude of the difference between the reference and the object increases. When this happens, the observer estimates that the object is closer in size to the reference than it actually is. As a result, an object smaller in size than the reference will be overestimated and an object larger will be under-estimated. This perfectly normal perceptual bias affects judgments of one’s own body size just as much as another person’s. It means that a plot of the body size one thinks one has (*y*-axis, in BMI units) as a function of one’s actual body size (*x*-axis, in BMI units) has a slope less than one: people with a BMI less than the population average will overestimate their size, those with a BMI close to the population average will be relatively accurate, and those with a BMI greater than the population average will under-estimate their size. In a 2D plot of this relationship, the location where the regression of self-estimated body size on actual body size intersects the *y*-axis is also controlled by an individual’s psychological concerns. Therefore, for any actual BMI, a given increase in body dissatisfaction will lead to the same increase in estimated body size. Typically, in our research, we have measured a range of psychological concerns, such as the participants’ attitudes toward their body shape/size, weight, and eating, as well as their tendency toward depression, and their self-esteem using psychometric measures. These measures have included the: Body Shape Questionnaire (BSQ-16b; Evans and Dolan, 1993), Eating Disorders Examination Questionnaire (EDE-Q; Fairburn and Beglin, 1994), Beck Depression Inventory (BDI; Beck et al., 1961), and Rosenberg Self-Esteem Scale (RSE; Rosenberg, 1965).

### Summary

To test these two hypotheses, we asked a sample of women with wide variation in both their BMI and psychological profiles to estimate both their own body size and the position of the “normal/overweight” categorical boundary for another woman, in an online study. The two hypotheses predict different patterns of responses, and the results will clarify the pressures that shape body size judgments.

## Materials and Methods

### Sample Size

To estimate a sample size appropriate to test hypothesis 1, we based our calculations on the high-level adaptation study conducted by Glauert et al. (2009). Prior to the adaptation phase of their protocol, women who varied on a measure of body dissatisfaction rated a range of bodies for how normal and ideal they looked. With respect to the normal ratings, when participant’s BMI was controlled for, their body shape concerns (measured with the body shape questionnaire, BSQ-34) were significantly negatively related to the BMI of the stimulus images that participants rated as most normal, *r* = −0.43, *p* < 0.002, giving an r^2^ of 0.18. For the purposes of a sample size estimation to test hypothesis 1, we assume that a “normal/overweight” boundary would be highly correlated with the location of the normal body size judgments in Glauert et al. (2009). Accordingly, on an F-test for a fixed regression model of normal body size on BSQ-34, a sample of 52 women would be required to return a power of 0.9 at an alpha of 0.05 (G*Power, v3.1.9.6).

To estimate a sample size appropriate to test hypothesis 2, we assume that the slopes of the multiple regression model predicting the “normal/overweight” boundary from participants’ body dissatisfaction and actual BMI will be very similar to those for predicting self-estimates of own body size. Irvine et al., (2018) used a method of adjustment task to obtain self-estimates of body size from 100 women, and also measured their body satisfaction with the BSQ-16 and actual BMI. An ordinary least squares (OLS) model with these two predictors explained 66.76% of the variance in self-estimates of body size. The unique variance explained by BMI and BSQ-16, respectively, was 0.384 and 0.0426. Therefore, to estimate a sample size for hypothesis 2 in the current study, we assumed an OLS multiple regression model with the same predictors, but powered the calculation (a fixed model increase in r-square) based on the smaller contribution to the model by BSQ-16. This rendered a sample size of 102 women to give a power of 0.9 at an alpha of 0.05 (G*Power, v3.1.9.6).

The sample size estimate to test hypothesis 2 (i.e., *n* = 102) exceeds that for hypothesis 1 (i.e., *n* = 52), therefore we selected a minimum sample size estimate of 102 for this study. However, the current study was run online, where it is not possible to ascertain how accurately and precisely participants’ height and weight are reported, and where we expect a high attrition rate because of the number of tasks participants were asked to perform. Therefore, we took a very conservative approach to the final sample size. Based on the power calculations above, we aimed to collect at least 120 to 130 datasets where participants had completed all tasks.

### Participants

This study depended on capturing individual variation in biometric, psychometric, and psychophysical performance in an opportunity sample of adult women. Therefore, we did not apply exclusory criteria when recruiting participants, beyond a requirement to read English. Advertisements for the study contained an anonymous link to the Qualtrics survey website (Qualtrics, Provo, UT) and were distributed through social media accounts belonging to four of the authors (LGB, JG, EL, and KRI). This allowed us to recruit 129 participants from the United Kingdom, Poland, Norway, and the Czech Republic, all of whom completed all questionnaires and psychophysical tasks. These individuals self-reported being assigned female at birth and being at least 18 years old. 86.05% of the 129 identified as White/Caucasian, 3.10% Asian, 3.10% Black/African American, 0.78% Arabic, 5.43% Hispanic/Latino, 1.55% Mixed/Other. Participant characteristics for the 129 complete psychometric/anthropometric data are described in Table 1.

**Table 1:** Characteristics of participants.

	*M*	SD	Range	
			Actual	Potential
Chronological age (yrs)	22.71	6.69	18.00–53.00	
Weight (kg)	67.45	15.38	43.00–112.00	
Height (cm)	166.12	7.70	133.00–193.00	
BMI	24.48	5.57	15.78–44.78	
EDE-Q Global	2.21	1.45	0.00–5.75	0–6
EDE-Q res	1.70	1.60	0.00–6.00	0–6
EDE-Q eat	1.47	1.32	0.00–5.00	0–6
EDE-Q sc	2.95	1.69	0.00–6.00	0–6
EDE-Q wc	2.73	1.78	0.00–6.00	0–6
BSQ-16	49.26	20.90	16.00–96.00	16–96
RSE	15.87	6.39	0.00–30.00	0–40
BDI	15.73	11.99	0.00–48.00	0–63

### Materials

#### Stimuli

Sixty-four Stimuli were selected from the database of 160 CGI (computer-generated imagery) images of a standard female model as described in Cornelissen et al. (2017). The woman stands in three-quarter view, is dressed in sports underwear, and her BMI ranges from 12.5 to 44.5 in 0.5 BMI steps. The images were created with DAZ v4.8 and were calibrated for BMI, based on the waist and hip circumference data from the Health Survey for England (Health Survey for England, 2003, 2009, 2012). They were rendered using Luxrender.^1^ The advantages of this stimulus set are that the images: (a) are high definition and photorealistic, (b) maintain the identity of the female model across a wide BMI range, and (c) demonstrate extremely realistic changes in BMI dependent body shape.

#### Psychometric and Anthropometric Measures

We administered a set of well-established, validated, self-report questionnaires to assess participants’ attitudes toward their body shape/size, weight, and eating, as well as their tendency toward depression, and their self-esteem. The following questionnaires were used:

The Eating Disorders Examination Questionnaire (EDE-Q; Fairburn and Beglin, 1994) is a self-report version of the Eating Disorders Examination (EDE) interview. The questionnaire contains four subscales: (a) the Restraint (EDE-Q res) subscale contains 5 items which measure the restrictive nature of eating; (b) the Eating Concern (EDE-Q eat) subscale contains 5 items which measure the preoccupation with food and social eating; (c) the Shape Concern (EDE-Q SC) subscale contains 8 items which measure dissatisfaction with body shape; (d) and the Weight Concern (EDE-Q WC) subscale contains 5 items which measure dissatisfaction with body weight. Participants report how many days of the past 4 weeks they have experienced an item, for example, “Have you been deliberately trying to limit the amount of food you eat to influence your shape or weight (whether or not you have succeeded)?” on a 7-point response scale from 0 indicates (no days) to 6 (every day). A global score of overall disordered eating behavior is also calculated by averaging the four subscales, and frequency data on key behavioral features are recorded. Cronbach’s alpha for this measure was 0.96 across all participants.

The 16-item Body Shape Questionnaire (BSQ-16b; Evans and Dolan, 1993) was used to assess size and shape concerns, for example, “Have you been so worried about your shape that you have been feeling you ought to diet?” Items are rated along a 6-point response scale, from 1 (never) to 6 (always). Items are summed for a total score. Cronbach’s alpha for this measure was 0.97 across all participants.

The Beck Depression Inventory (BDI; Beck et al., 1961) was used to measure levels of depressive symptomatology. It is a behavioral and attitudinal checklist that contains 21 items, such as “loss of interest,” “sadness,” and “self-dislike.” Each item is rated on a 4-point scale, ranging from 0 (no symptom of depression) to 3 (severe expression of a depressive symptom). Items are summed for a total score. Cronbach’s alpha for this measure was 0.94 across all participants.

The Rosenberg Self-Esteem Scale (RSE; Rosenberg, 1965) was used to assess self-esteem by reflection on current feelings. The 10 items are rated on a 4-point scale from “strongly disagree” to “strongly agree.” Five of the items have positively worded statements, for example, “On the whole I am satisfied with myself” and five are worded negatively, for example, “At times I think that I am no good at all.” Items are summed for a total score. Cronbach’s alpha for this measure was 0.92 across all participants.

Participants’ body mass index (BMI) was calculated from their self-reported weight and height. On screen, they were shown a sequence of graphic images to illustrate how to measure their height and weight with accompanying instructions: (a) “please remove any footwear and stand straight against a wall or flat surface. Then temporarily mark your height, preferably with a line, from the top of your head. Finally, measure the distance from the ground to the mark to measure your height,” and (b) “please remove shoes and heavy clothes, then weigh yourself using a scale.”

#### Psychophysical Measures

The Method of Adjustment (MoA) task was created using the PsychoJS JavaScript library, which is part of PsychoPy3 (Peirce et al., 2019). The psychophysical aspects of the study were hosted online on pavlovia.org, which handled the storage and delivery of the necessary web scripts, URL, and subsequent data storage. The survey platform (Qualtrics) randomly assigned the presentation order of the two experimental conditions and sent this information to the psychophysical task *via* a query string embedded in the URL. The task needed to be completed using a desktop browser (i.e., not a tablet or mobile phone) and was always presented full screen. The software was designed to identify the platform used, and politely requested participants to use a desktop or laptop PC in the event that a tablet or mobile phone was detected.

The same MoA task was used for the two experimental conditions: (a) participants making self-estimates of their own body size, and (b) judging when another woman’s body has just changed from being normal size to overweight. The only difference between conditions was the initial instructions before the task began, and the wording of the task reminder on every trial of the task.

**Figure 1:**
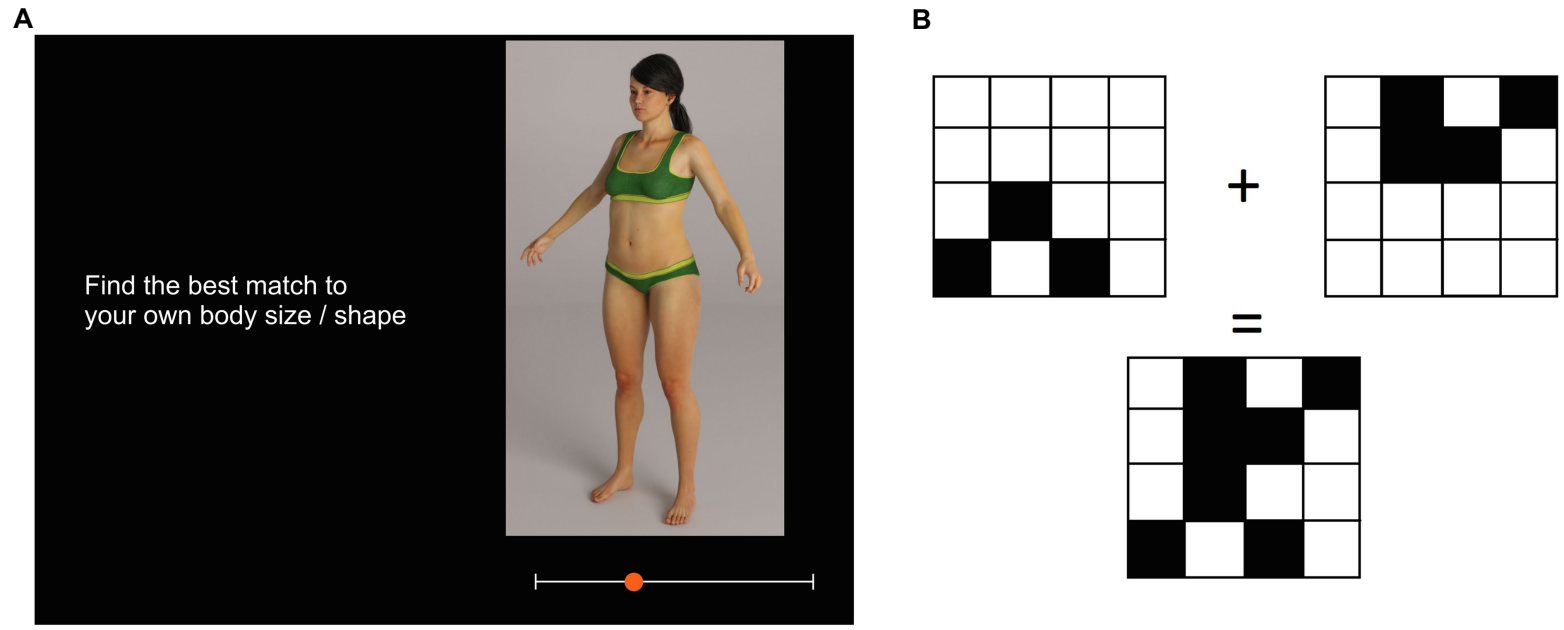
Schematics to illustrate: **(A)** The appearance of the stimulus, response slider, and task reminder on one trial of the MoA for self-estimation of body size, and **(B)** the appearance of the stimuli on one trial of the distractor task.

Each condition comprised 20 trials. At the start of each trial, a white plus sign appeared in the middle of a black screen on which participants had to click with their mouse pointer. This was replaced by: (a) a task reminder on the left of the screen (i.e., “Find the best match to your own body size/shape” or “Find where the woman just changes from normal size to overweight, in your opinion”); (b) a stimulus image on the right side of the screen (scaled relatively to 80% of the devices screen height while maintaining the original image aspect ratio); and (c) a white horizontal scale bar with a circular red button overlying it (scaled relatively to approximately 33% of the screen width), at the bottom of the screen (See Figure 1A). Participants were asked to click on the red button and drag it to a new location on the scale bar to change the size of the avatar. If the red button was dragged to the extreme left of the scale bar, the avatar shrank to her lowest BMI. If the red button was dragged to the extreme right of the scale bar, the avatar expanded to her highest BMI. On each trial, participants were asked to move the button as many times as it took them to find a match between the avatar’s size and the size they sought for the particular task, at which point they pressed the space bar. This saved the BMI of the image that participant’s chose as a response to file and initiated the next trial. The task prohibited participants from moving on without interacting with the slider at least once per trial. The horizontal location of the stimulus image was jittered horizontally from one trial to the next to prevent participants using spatial cues to remember the location of the red button in relation to the stimulus. In addition, the initial appearance of the avatar and the red button was randomized between its lowest and highest BMI settings from one trial to the next. The order in which participants carried out the two conditions for the MoA was alternated between successive participants. Critically, participants also carried out a distractor task between each of the MoA conditions, to minimize any carry over between the two kinds of body size judgment. The extent that participants forget the content of a previous task depends on the difficulty of the subsequent intervening task (Bjork and Allen, 1970; Roediger and Crowder, 1975). Therefore, to achieve this, we used a short but highly taxing working memory task, the visuo-spatial n-back task.

#### Distractor Task

The n-back task comprised 15 trials. On each trial, on a white background, participants were presented three 3 × 3, 4 × 4, or 5 × 5 grids of squares. One grid appeared to the upper left quadrant of the screen, one to the upper right quadrant, and one in the midline of the screen below the bottom of the first two. In addition, a plus sign appeared between the upper left and upper right grids, and an equals sign just above the third grid (See Figure 1B). An arbitrary number of the squares in each grid were blacked out, and the participants’ task was to decide whether the grid at the bottom of the screen represented the sum of the first two. Participants had to respond “yes” or “no” by key press. The distractor task is precisely that: it was intended to minimize cross-contamination between the two MoA tasks. The results were not subsequently used in the study.

### Procedure

Once participants clicked on the link to Qualtrics, they were presented a description of the study, which gave them enough information to consent to take part. By this stage, the program had detected the platform that the participant was using and politely reminded them that to complete the survey they would have to use a laptop or desktop PC, rather than a mobile phone or tablet. After this, the participant was required to provide demographic information, their height, and weight. They then were asked to complete the four psychometric questionnaires: EDE-Q, RSE, BDI, and BSQ-16. At this stage, participants were automatically redirected to Pavlovia.org and were asked to wait while the images for the two MoA tasks and the distractor tasks were uploaded. Once the psychophysical and distractor tasks were complete, participants were directed back again to Qualtrics and were presented with the study debrief. This entire procedure took approximately 30 minutes to complete.

Note that the body size women believe they have, and the location of the “normal/overweight” boundary that observers set, were both calculated offline as the average BMI of the images chosen at the end of the 20 trials, separately for each of the two MoA tasks.

## Results

### Univariate Statistics

Participant characteristics are described in Table 1. Overall, these data suggest that, on average, the women who successfully completed this study had mild concerns about their bodies, coupled with a tendency for lower self-esteem and mild depressive symptomatology. Nevertheless, consistent with study requirements, we found wide variation in biometric, psychometric and psychophysical performance.

### MoA Split-Half Reliability

On each of the 20 trials in the MoA tasks, we recorded the BMI of the image that participants’ chose on each trial, as well as the amount of time it took for them to make a response. The response times (RT) were positively skewed, and therefore transformed logarithmically. Table 2 shows the mean BMI response and log_10_RT for the first 10 trials and the second 10 trials, separately for self-estimated body size and the “normal/overweight” boundary judgments.

**Table 2:** Split-half reliability analysis of MoA data.

Condition	Trials	BMI		Log_10_ RT	
		Mean	SD	Mean	SD
Self-estimated body size	1–10	25.20	6.28	0.73	0.41
	11–20	25.01	6.07	0.48	0.35
“Normal/overweight” boundary	1–10	28.56	5.49	0.80	0.41
	11–20	29.06	5.47	0.56	0.35

We used PROC MIXED (SAS v9.4) to run separate linear mixed effects models of response BMI and log_10_ RT, including experimental condition (i.e., self-estimates of body size and “normal/overweight” boundary) and trial block (i.e., trials 1–10 and 11–20) as explanatory variables. A random effect was included for participant intercept in each model. For response BMI, we found a statistically significant fixed effect of condition (*F*1,384 = 94.05, *p* < 0.0001) but not for trial block (*F*1,384 = 0.16, *p* = 0.69). There was no significant interaction between condition and trial block (*F*1,384 = 0.83, *p* = 0.36). For log_10_ RT, we found a statistically significant fixed effect of condition (*F*1,384 = 21.88, *p* < 0.0001) and trial block (*F*1,384 = 202.45, *p* < 0.0001). There was no significant interaction between condition and trial block (*F*1,384 = 0.03, *p* = 0.85). Post-hoc pairwise comparisons of LSmeans showed statistically significant reductions in log_10_ RT between trials 1–10 and 11–20 for both the “normal/overweight” boundary task (*t*384 = 9.93, *p* < 0.0001), and self-estimates of body size (*t*384 = 10.19, *p* < 0.0001). Finally, the Pearson correlations for BMI responses between trials 1–10 and 11–20 for self-estimates of body size and the “normal/overweight” boundary task were, respectively: *r* = 0.98, *p* < 0.0001 and *r* = 0.97, *p* < 0.0001.

These data suggest that within each MoA task, the data are reliable. However, while participants took longer to respond in the first half of each MoA task than the second half, it is also clear that participants took longer to respond overall in the “normal/overweight” boundary task compared to their self-estimates of body size. This suggests that the “normal/overweight” boundary task may have either have been more difficult and/or required more cognitive resources.

### Self-Estimated Body Size

Prior to multivariate analysis, Shapiro–Wilk tests showed that self-estimated body size, chronological age, actual BMI, EDE-Q, BSQ-16, and BDI did not conform to normal distributions (*W* = 0.91, *p* < 0.0001; *W* = 0.62, *p* < 0.0001; *W* = 0.88, *p* < 0.0001; *W* = 0.95, *p* = 0.0002; *W* = 0.96, *p* = 0.0008; *W* = 0.93, *p* < 0.0001, respectively). Therefore, these variables were logarithmically transformed.

In our first analysis, we wanted to test whether we could replicate the findings of Cornelissen et al. (2015, 2016, 2017) and Irvine et al. (2018). Specifically, we wanted to confirm whether a regression of self-estimated body size (log_10_BMI units) on actual body size (log_10_BMI units) showed: (a) evidence of contraction bias, that is, a slope less than 1 with a rotation point around the average BMI for women, and (b) an independent contribution to estimated body size from participants’ psychometric performance. To avoid the possibility of introducing substantial variance inflation, we first checked for evidence of co-linearity among the psychometric variables.

**Figure 2:**
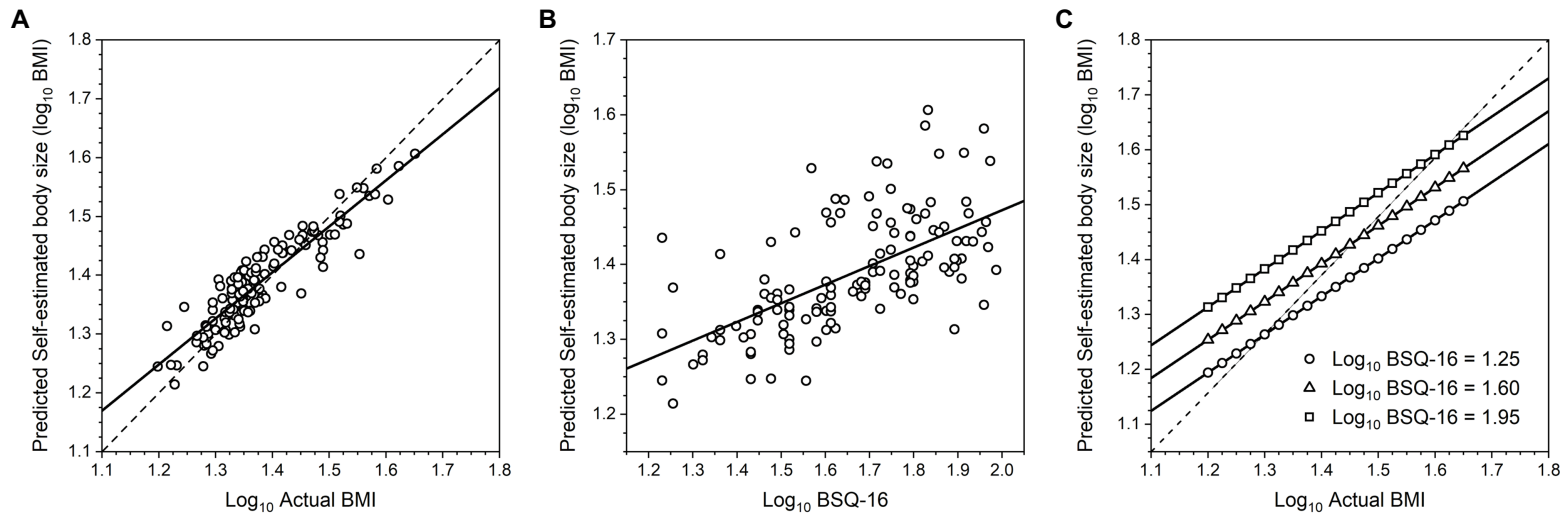
**(A)** Scatter plot of log_10_ self-estimated body size as a function of log_10_ actual BMI, predicted from the multiple regression model. The dashed line represents the line of equivalence, that is, where participants’ estimates would exactly match their actual BMI, and this line has a slope of 1. The solid line represents the regression of log_10_ self-estimated body size on log_10_ actual BMI across the whole sample, and this has a slope less than 1. **(B)** Scatter plot of log_10_ self-estimated body size as a function of log_10_ BSQ-16, predicted from the multiple regression model. **(C)** Graphical illustration of the multiple regression of log_10_ self-estimated body size on log_10_ actual BMI, at three levels of log_10_ BSQ-16, corresponding to BSQ-16 scores of ~18, ~40, and ~ 90. This graph therefore illustrates: **(A)** there is evidence for contraction bias across the entire sample, and **(B)** at any actual BMI, increasing BSQ-16 increases self-estimates of body size independently.

Given that Table 3 shows substantial and significant Pearson correlations between log_10_ EDE-Q, log_10_ BSQ-16, RSE, and log_10_ BDI, we sought to include a selection procedure for the model that would avoid potential problems with multicollinearity. Since stepwise selection algorithms are known to lead to biases in parameter estimation (Hurvich and Tsai, 1990; Steyerberg et al., 1999; Grafen and Hails, 2002), we used PROC GLMSELECT in SAS v9.4 (SAS Institute, North Carolina, United States) to run adaptive LASSO (least absolute shrinkage and selection operator) regression for variable selection (Tibshirani, 1996; Osborne et al., 2000; Efron et al., 2004). LASSO and stepwise regression differ in their criteria for retaining predictors in the final model, and LASSO has been shown to produce more stable results. The LASSO algorithm selects an optimal value for *t*, the tuning or shrinkage parameter which, in our case, minimized the Schwarz Bayesian information criterion (SBIC) for model fitting. We included log_10_ chronological age, log_10_ actual BMI, log_10_ EDE-Q, log_10_ BSQ-16, RSE, and log_10_ BDI as explanatory variables at the start of the selection procedure. By the end of selection, the optimal subset of variables chosen to model self-estimated body size had a minimum SBIC value of −711.51. We then used PROC REG in SAS (v9.4) to run ordinary least squares multiple regression models with this reduced set of explanatory variables (i.e., log_10_ BMI and log_10_ BSQ-16), derived from the LASSO process, and where we also tested for the presence of significant interaction terms. The final model explained 62.5% of the variance in self-estimated body size, the slope of the regression of self-estimated body size on log_10_ actual BMI was significantly less than 1 [*F*(1,126) = 24.94, *p* < 0.0001], and the regression line crossed the line of equivalence (see Figure 2A) at an actual BMI of ~26 (i.e., log_10_ actual BMI = 1.42). We found no evidence for statistically significant interaction terms in the model. Table 4 shows the model parameters (Model 1: Self-estimated body size), and Figure 2 is a graphical illustration of the model outcomes.

**Table 3:** Pearson correlations between psychometric variables.

	log_10_ EDE-Q	log_10_ BSQ-16	RSE
log_10_ BSQ-16	0.88^***^	–	
RSE	−0.55^***^	−0.58^***^	–
log_10_ BDI	0.62^***^	0.64^***^	−0.75^***^

* *p* < 0.05;

** *p* < 0.01;

*** *p* < 0.0001.

**Table 4:** Outputs from the multiple regression models.

Model	Parameter	*t* (*DF*)	*p*-value	Estimate	*95% CI*
1) Log_10_ S-E	Intercept	1.71(1)	0.09	0.15	−0.024–0.32
	Log_10_ aBMI	11.36(1)	<0.0001	0.69	0.57–0.82
	Log_10_ BSQ-16	5.98(1)	<0.0001	0.17	0.11–0.23
2) N/O boundary	Intercept	4.60(1)	<0.0001	38.40	21.90–54.91
	Log_10_ age	−2.73(1)	0.007	−12.62	−21.78–3.46
	Log_10_ S-E	3.65(1)	0.0004	19.20	8.79–29.61
	Log_10_ BSQ-16	−4.35(1)	<0.0001	−11.67	−16.97–6.36

Log_10_ S-E, Log_10_ self-estimated body size; N/O boundary, “Normal/overweight” boundary; Log_10_ aBMI, Log_10_ actual BMI.

### Estimates of the “Normal/Overweight” Boundary in Others

**Figure 3:**
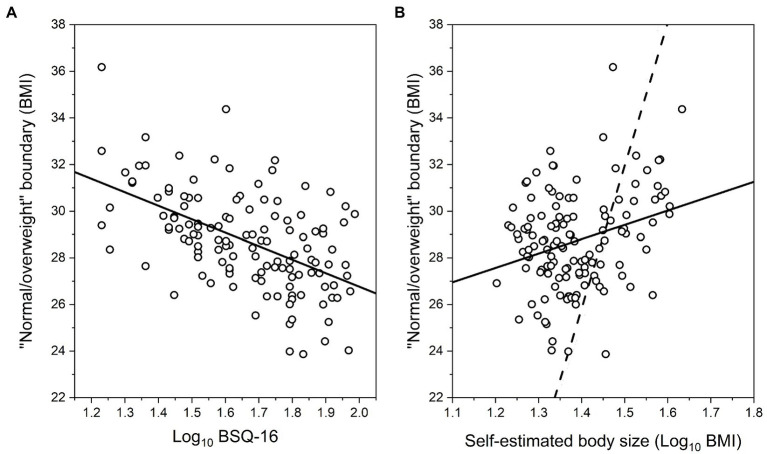
Scatter plots of predicted “normal/overweight” boundary judgments as a function of: **(A)** log_10_ BSQ-16, and **(B)** log_10_ self-estimated body size, from the multiple regression model. Each case shows the regression lines through the data (solid). The dashed line in **(B)** represents matched responses, that is, where participants’ “normal/overweight” boundary judgments would exactly match their estimates of their own body size.

Our first hypothesis predicts that: (a) the size of the “normal/overweight” boundary in another woman should *reduce* as observers’ body dissatisfaction (indexed by psychometric task performance) increases, and (b) there should be no relationship between this boundary and observers’ actual body size. Our second hypothesis predicts that the “normal/overweight” boundary should be directly related to the size that someone believes themselves to be. Therefore, we again used PROC GLMSELECT in SAS v9.4 (SAS Institute, North Carolina, United States) to run an adaptive LASSO regression to select the minimum number of explanatory variables needed to explain variance in the “normal/overweight” boundary task. We included log_10_ chronological age, log_10_ self-estimates of body size, log_10_ actual BMI, log_10_ EDE-Q, log_10_ BSQ-16, BAS, log_10_ BDI, and RSE as explanatory variables at the start of the selection procedure. By the end of selection, the optimal subset of variables chosen to model performance in the “normal/overweight” boundary task had a minimum SBIC value of 426.64. We then used PROC REG in SAS (v9.4) to run an ordinary least squares multiple regression model with this reduced set of explanatory variables (i.e., log_10_ chronological age, log_10_ self-estimated body size, and log_10_ BSQ-16), derived from the LASSO selection procedure, and where we also tested for the presence of significant interaction terms. The final model explained 15.75% of the variance in the “normal/overweight” boundary task, and the model parameters are shown in Table 4 (Model 2: Normal/overweight boundary). We found no evidence for statistically significant interaction terms. To illustrate the outcome, Figures 3A,B show plots of predicted “normal/overweight” boundary judgments as a function of log_10_ BSQ-16 and log_10_ self-estimated body size, respectively.

## Discussion

This study explored the relationships between our female participants’ estimates of their own body size, their subjective judgments about when another woman’s body just starts to appear overweight, and their own level of body dissatisfaction. We proposed two hypotheses for what these relationships might be. In the first hypothesis, participants compare the image of the woman presented on screen with their internalized version of the Western “thin ideal.” For each participant, we proposed that the size of their internalized “thin ideal” will be inversely proportional to their degree of body dissatisfaction and be independent of their actual body size. Consequently, as their own body size dissatisfaction increases, so the body size of the “thin ideal” shrinks, as does the size at which that ideal can be described as overweight. *Thus, we predicted an inverse relationship between the “normal/overweight” boundary and the participants’ own body dissatisfaction*. The second hypothesis proposed that a participant judges the “normal/overweight” boundary for another woman in the context of the size that they think their own body has. Because this represents a direct comparison between one’s self and someone else, this constitutes an explicit or implicit social comparison (Festinger, 1954). Given that the participant is free to select any body size to represent the “normal/overweight” boundary, we suggest that their choice will not trigger an upward social comparison (i.e., picking a slimmer body), since this could be distressing. Instead, we predicted that the participant should select a body size for the “normal/overweight” boundary in another woman that represents either a neutral comparison (i.e., the same size as they believe themselves to be) or a downward comparison, where the selected body size is larger than the size the participant believes themselves to have.

Our first concern was to check whether the regression of self-estimated body size on actual body size and psychometric performance showed statistically independent contributions from: (a) a perceptual contraction bias, where the slope of the relationship between self-estimated body size and actual BMI is less than 1, with a rotation point around the average BMI for women (see Figure 2A), and (b) an attitudinal component whereby, for any actual BMI, increasing psychological concerns about body shape, weight, and eating lead to larger body size estimates (see Figure 2B) (*cf.* Cornelissen et al., 2015, 2016, 2017; Irvine et al., 2018). Our first multivariate analysis does indeed confirm this, as shown in Table 4 (Model 1: Log_10_ Self-estimated size) and illustrated in Figure 2C.

With respect to our participants’ judgments of the “normal/overweight” boundary position, we found clear support for the first hypothesis, as illustrated in Table 4 (Model 2: Normal/overweight boundary) and Figure 3A, which show an inverse relationship between the “normal/overweight” boundary and the participants’ own body dissatisfaction, as indexed by their BSQ-16 scores, even when the chronological age of the participant is factored in. Moreover, the participants’ actual body size played no part in their judgments of the position of the “normal/overweight” boundary. With respect to our second hypothesis, Table 4 (Model 2: Normal/overweight boundary) and Figure 3B show very clearly that the size participants *believed* themselves to be played an independent, and statistically significant role in “normal/overweight” boundary judgments. However, this evidence does not map onto hypothesis 2 in a straightforward way. According to hypothesis 2, participants’ “normal/overweight” boundary judgments should parallel their self-estimated body size, which would mean that the slope of the regression of boundary judgments on self-estimated body size should be close to the line of equivalence (i.e., where a given self-estimated body size in BMI units predicts the *same* “normal/overweight” boundary for another person, in BMI units). But, as Figure 3B shows, while we found a positive regression slope, the gradient is less steep than the line of equivalence (i.e., the dashed line in Figure 3B). In practice, what this means is “normal/overweight” boundary judgments were greater than self-estimated body size up to ~28 BMI units (i.e., log_10_ BMI = 1.45). However, above this BMI value, “normal/overweight” boundary judgments were lower than self-estimated body size. Therefore, either hypothesis 2 is wrong, or it needs to be modified to accommodate this result. We know that when healthy female observers judge the weight (in kilograms or stones) of other women displayed in photographs, then we observe a contraction bias between the observers’ responses and the known weights of the women in the photographs (Cornelissen et al., 2016). Photographs of women with a body weight which is less than the population average are overestimated, women whose body weight is closest to the population average are most accurately judged, and women whose body weight is greater than the population average are under-estimated. In the current study, we know from Table 4 (Model 1: Self-estimates of body size) and Figure 2 that there is a contraction bias between participants’ actual BMI and the body size they believe they have. Therefore, one way to modify hypothesis 2 would be to suggest that there is an additional contraction bias between the size that a woman thinks she is and the size of the woman on screen, in the context of making a neutral or downward social comparison to select the “normal/overweight” boundary.

### Possible Mechanism for How Family/Peer Pressure May Trigger Body Dissatisfaction

Family can play an important role in developing concerns about body weight and size (Kluck, 2010; Hardit and Hannum, 2012). There seems to be a significant relationship between familial criticism, teasing, and encouragement about weight or size with body dissatisfaction (Kluck, 2010). Additionally, there is potentially a strong effect of sibling and peers with whom they may be more likely to compare their own appearance as they closer in age and will have the most day-to-day contact (e.g., Lev-Ari et al., 2014).

We suggest that the present results offer one mechanism by which peer/family pressure may operate. Essentially, if a peer or family member experiences attitudinal body dissatisfaction for themselves, then they may internalize an unusually thin version of the “thin ideal.” For example, from Figure 3A, if such an individual has no concerns with their own body shape, that is, a BSQ-16 score ~ 20 (log_10_ BSQ-16 = 1.3), this predicts a “normal/overweight” boundary ~30 BMI units which corresponds to the World Health Organization (WHO) category boundary for obesity. However, if an individual has marked concerns about their own body shape, that is, a BSQ-16 score of ~85 (log_10_ BSQ-16 = 1.9), this predicts that they would apply a “normal/overweight” boundary at around ~26.5 BMI units to another woman. This therefore raises the possibility that such a parent or peer may start to criticize someone’s body size at a much lower BMI threshold, with the attendant risk of triggering body image discontent in the recipient of the criticism. For example, by making disparaging remarks about one’s body, and/or that of others’ (“fat talk”), which has been well-established as a risk factor to body image issues (for meta-analysis see Mills and Fuller-Tyszkiewicz, 2017). Consistent with this interpretation, Bauer et al. (2013) investigated cross-sectional relationships between parental weight talk, as reported by mothers, and a wide range of outcomes for their daughters, including depression, use of weight control behaviors, and prevalence of binge eating. Bauer et al. (2013) found that more frequent comments to daughters about their weight were associated with greater prevalence for all three of these negative outcomes, even after adjustment for socio-demographic characteristics and girls’ standardized BMI. Recently, comparable results were reported for the interactions between boys and their mothers, by Solano-Pinto et al. (2021).

### Limitations and Future Research

#### Self-Estimates of Body Size

In this study, we relied on our participants to report their height and weight and we could not independently verify the accuracy of their reports. The same problem has been encountered in many epidemiological studies of population rates for overweight and obesity, where it is known that participants tend to overestimate height, and under-estimate weight, leading to under-estimates of BMI. To counteract this, a number of research groups have developed correction techniques, based on datasets where both measured and self-estimated height and weight are available (see, e.g., Gorber et al., 2008; Dutton and McLaren, 2014; Drieskens et al., 2018). We applied the approach developed by Dutton and McLaren (2014) to the current study, but this only increased the variance in self-estimates of body size explained by the model from 62.5 to 62.6%. Almost certainly, this is because these corrections are designed to shift the location and width of a measured BMI distribution, while retaining the same relative ranking of individual body weights/heights. Clearly, this will be effective in terms of calculating what proportion of a sample exceed a given BMI threshold, comparing the original to the corrected distributions. However, we suspect that the “noise” in our data may be better characterized as a change in the relative ranking of body weights and heights across the sample, for which these approaches to correction will not be effective. For example, for those who did measure their own weight in the current study, there will random fluctuation in the accuracy of weighing scales across different households, with some under-reporting and others over-reporting weight. In support of this argument is that fact that Irvine et al. (2018) asked 100 healthy adult females to estimate their body size using a laboratory-based MoA task. The regression model they report used actual BMI, derived from calibrated height and weight measurements obtained from the same equipment, and BSQ-16 as explanatory variables. It accounted for 67.0% of the variance in self-estimated body size. By comparison, in the current online study, an equivalent analysis explained a smaller, albeit similar proportion of the variance (i.e., 62.5%). It would therefore be reassuring to repeat this study in the laboratory, where one has full control over the height and weight measurements of participants, to seek a replication. Moreover, in a laboratory setting, one would ideally obtain psychophysical estimates from two techniques: for example, the method of adjustment, as we used, as well as a forced choice task in combination with the method of constant stimuli (Gescheider, 1997).

#### Alternative Potential Sources of Variation in the “Normal/Overweight” Boundary

One potential limitation is that we did not provide a definition of, or measure how participants interpreted the word “overweight” in the “normal/overweight” boundary task. It is possible that some participants may have seen it as a value judgment, rather than a neutral descriptor of adiposity. Due to the social presence of the thin ideal in the Western world, which values thinner bodies over heavier bodies, “overweight” to some extent may be used as a value judgment instead of a neutral descriptor of size. This is illustrated by studies which suggest a prevalence of anti-fat bias, which is the negative attitude toward, belief about, or behavior against people perceived as being “fat” (Danielsdottir et al., 2010) and is believed to arise from the adoption of the thin ideal (Crandall and Schiffhauer, 1998). Moreover, there is evidence for: (a) varying levels of both implicit and explicit anti-fat bias in both clinical (Cserjési, et al., 2010; Spring and Bulik, 2014) and non-clinical populations (Klaczynski et al., 2004; Puhl et al., 2007), and (b) positive linkage to body image distortion scores (Lydecker et al., 2019) and overall thin idealization (Thompson and Stice, 2001; Dittmar, and Howard, 2004; Brown and Dittmar, 2005; Fitzsimmons-Craft et al., 2012). Thus, individuals with high levels of anti-fat bias might well interpret “overweight” in a body image context as a more negative judgment compared to an individual with lower levels of anti-fat bias, and this could introduce a source of variation into the data that we have not quantified.

Our study assumes that our participants had internalized the thin cultural ideal but we did not explicitly test for internalization *per se*. Nevertheless, consistent with this assumption, we found that participants with high BSQ-16 scores tended to overestimate their own size which arguably implies some level of internalized weight bias/thin idealization. Therefore, future research may benefit from measuring the degree to which internalization occurs in participants and any potential interaction effects these variables may have on the relationships between self-estimated body size, own BSQ-16 scores, and the “normal/overweight” boundaries for other women. Furthermore, future studies should also index the degree to which participants are likely to make social comparisons when judging body size and the relative importance they place on these comparisons and whether this also modulates the boundary. In addition, it may be beneficial to be more specific about which definition of “overweight” we want participants to use, and have participants perform the “normal/overweight” boundary task twice: once where they are asked to choose where another woman’s body becomes overweight, and a separate task where they are asked at what size their own body becomes overweight. It might also be informative to ask participants to choose their ideal body size, and then use this as a reference point instead of where they think the normal/overweight boundary falls. This would also allow a measure of body dissatisfaction (the difference between actual and ideal body size) to be calculated. The addition of the ideal estimation was not included in the current study due to time constraints on what was already quite a long experiment.

### Conclusion

In conclusion, we found that women’s judgments about when someone’s body starts to be categorized as overweight can be explained by two opposing factors. Increasing body dissatisfaction in the observer leads to slimmer judgments for the position of the “normal/overweight” boundary in the BMI spectrum. In contrast, increasing overestimation by the observer of their own body size leads to shift toward higher BMI levels for the position of the boundary.

## Data Availability Statement

The raw data supporting the conclusions of this article will be made available by the authors on request.

## Ethics Statement

The studies involving human participants were reviewed and approved by Department of Psychology Ethics Committee, Northumbria University. The patients/participants provided their written informed consent to participate in this study.

## Author Contributions

KC: conceptualization, funding acquisition, methodology, project administration, resources, supervision, writing—original draft, and writing—review and editing. LB, JG, and EL: investigation, methodology, writing—original draft, and writing—review and editing. KM: methodology, software programming, and writing—review and editing. KRI: investigation, methodology, and writing—review and editing. MT: conceptualization, methodology, writing—original draft, and writing—review and editing. PC: conceptualization, data curation, formal analysis, methodology, validation, visualization, roles/writing—original draft, and writing—review and editing. All authors contributed to the article and approved the submitted version.

## Funding

LB was funded by Northumbria University as a Research Assistant for this project.

## Conflict of Interest

The authors declare that the research was conducted in the absence of any commercial or financial relationships that could be construed as a potential conflict of interest.

## Publisher’s Note

All claims expressed in this article are solely those of the authors and do not necessarily represent those of their affiliated organizations, or those of the publisher, the editors and the reviewers. Any product that may be evaluated in this article, or claim that may be made by its manufacturer, is not guaranteed or endorsed by the publisher.

## Footnotes

^1^ https://luxcorerender.org/
